# Autoantibody profiling of patients with immune checkpoint inhibitor-associated myocarditis: a pilot study

**DOI:** 10.3389/fimmu.2024.1423622

**Published:** 2024-09-11

**Authors:** Siqi Li, DongZhu Xu, Nobuyuki Murakoshi, Zixun Yuan, Takuro Imaoka, Kazuko Tajiri

**Affiliations:** ^1^ Department of Cardiology, Institute of Medicine, University of Tsukuba, Tsukuba, Japan; ^2^ Tsukuba Life Science Innovation Program (T-LSI), School of Integrative and Global Majors (SIGMA), University of Tsukuba, Tsukuba, Japan; ^3^ Stanley and Judith Frankel Institute for Heart & Brain Health, University of Michigan Medical School, Ann Arbor, MI, United States; ^4^ Department of Cardiology, National Cancer Center Hospital East, Kashiwa, Japan

**Keywords:** cardio-oncology, onco-cardiology, immune-related adverse event, irAE, proteome, proteomics, autoimmunity

## Abstract

**Background:**

Immune checkpoint inhibitor (ICI)-associated myocarditis is a rare, but potentially fatal, immune-related adverse event. Hence, identifying biomarkers is critical for selecting and managing patients receiving ICI treatment. Serum autoantibodies (AAbs) in patients with ICI myocarditis may serve as potential biomarkers for predicting, diagnosing, and prognosing ICI myocarditis. We conducted a pilot study using a human proteome microarray with approximately 17,000 unique full-length human proteins to investigate AAbs associated with ICI myocarditis.

**Methods and results:**

AAb profiling was performed using sera collected from three patients with ICI myocarditis before the start of ICI treatment and immediately after myocarditis onset. All patients received anti-programmed death-1 antibody monotherapy. At baseline, 116, 296, and 154 autoantigens reacted positively to immunoglobulin G (IgG) in the serum samples from Cases 1, 2, and 3, respectively. Among these proteins, the recombination signal-binding protein for the immunoglobulin kappa J region (RBPJ) was recognized by all three samples, and 32 autoantigens were recognized by any two of the three samples. At the onset of ICI myocarditis, compared to baseline, 48, 114, and 5 autoantigens reacted more strongly with IgG in the serum samples from Cases 1, 2, and 3, respectively. Among these, antibodies against eukaryotic translation initiation factor 4E binding protein 3 (EIF4EBP3) were the most upregulated, with a 38-fold increase. Gene ontology (GO) and Kyoto Encyclopedia of Genes and Genomes (KEGG) enrichment analyses highlighted that B-cell receptor signaling, leukocyte transendothelial migration, and thymus development were among the most affected pathways. Enrichment analyses using DisGeNET revealed that proteins reacting to AAbs detected in patients with ICI myocarditis are associated with several diseases, including dilated cardiomyopathy and muscle weakness.

**Conclusions:**

This pilot study provides the first integrated analysis of serum AAb profiling in patients with ICI myocarditis and identifies novel candidate markers associated with an increased risk of developing ICI myocarditis and its pathogenesis. However, our results require further independent validation in clinical trials involving a larger number of patients.

## 1 Introduction

Immune checkpoint inhibitors (ICIs) are a novel class of immunotherapeutic drugs that improve the treatment of a broad range of cancers. These drugs are increasingly being used for a large number of solid and hematological malignancies in the early stages, and several clinical trials are underway to expand their indications (1). However, the benefits of ICIs are often mitigated by the development of immune-related adverse events (irAEs) (2–4). In particular, myocarditis is recognized as a life-threatening complication with a mortality rate of up to 50% (5–7), but little is known about its immunological mechanisms and potential biomarkers.

In patients with classical myocarditis or inflammatory cardiomyopathy, autoantibodies (AAbs) against a wide range of self-antigens are detected. These AAbs can be specific or nonspecific to heart tissue, and several have been reported to be directly related to the pathophysiology (reviewed in (8, 9)). Immunoadsorption therapy has been performed to remove circulating AAbs in patients with inflammatory cardiomyopathy, resulting in an improvement in cardiac function and decreased myocardial inflammation in some small studies with a limited number of patients (10–12). However, reports on AAbs in patients with ICI myocarditis are limited. A recent study reported that the presence of anti-acetylcholine receptor (AChR) antibodies was more prevalent in patients with ICI myocarditis than in ICI-treated control patients. Among patients with ICI myocarditis, anti-AChR antibodies were associated with a higher incidence of cardiomyotoxic events (a composite of life-threatening arrhythmias, severe heart failure, severe respiratory muscle failure, or cardiomyotoxic death) (13). In addition to these findings, no comprehensive investigation on the presence of AAbs in patients with ICI myocarditis has been performed to date.

If AAbs associated with the development of ICI myocarditis are identified, screening could be useful for the diagnosis and prediction of ICI myocarditis. Moreover, treating ICI myocarditis with immunoabsorption or plasma exchange could be a valuable addition to the current standard corticosteroid therapy (14). Furthermore, AAb profiling may help further elucidate the pathogenesis of ICI myocarditis. Therefore, we conducted a pilot study to characterize AAb profiles in cancer patients with ICI myocarditis.

## 2 Material and methods

### 2.1 Clinical data and sample collection

This study included six serum samples from three patients with ICI myocarditis. Patients were enrolled in a prospective biospecimen collection protocol approved by the Ethics Committee of the University of Tsukuba Hospital (H30-221). Written informed consent was obtained from all patients. Clinical, radiographic, and laboratory data were collected from electronic medical records. Blood samples were collected before ICI treatment and at the onset of ICI myocarditis (before steroid therapy). The collected serum samples were aliquoted into smaller volumes and stored at −80°C until further use.

### 2.2 Serum autoantibody profiling

Comprehensive profiling of serum AAbs was conducted using HuProt™ Human Proteome Microarray v3.1 (CDI Laboratories, Inc, Baltimore, MD, USA), which contained about 17,000 unique proteins. One array per sample was used for the serum profiling. All serum samples were probed on the arrays at a 1:1000 dilution, as optimized by CDI labs, and incubated overnight at 4°C to enhance potential interactions. After probing, the arrays were washed according to the manufacturer’s protocol and probed with anti-human immunoglobulin G (IgG) antibodies optimized by the CDI laboratories for signal detection.

Non-specific hits that directly bound to the secondary antibody were eliminated from the analysis. The CDI software was used to quantify the specificity of each sample for specific proteins on the array based on Z-scores. The Z-score is the average Z-score of the duplicate spots of a given protein (each protein is printed in duplicate on a HuProt™ array). Z score was calculated as: *Z = [F_protein_ – F_average_]/F_SD_* where *F_protein_* is the fluorescence signal intensity of a specific protein, *F_average_* is the mean fluorescence signal intensity of all protein spots on the array, and *F_SD_* denotes the standard deviation of all protein spots on the array. Positive hits were defined as Z-scores > 3.0. AAbs that increased after the onset of ICI myocarditis were identified by following criteria: (1) statistical differences between baseline and after developing ICI myocarditis (*P* < 0.05), assessed using the Mann-Whitney *U* test; and (2) fold change (FC) ≥ 2.0.

Gene Ontology (GO), Kyoto Encyclopedia of Genes and Genomes (KEGG) functional, and DisGeNET disease enrichment analyses, based on Metascape software (https://metascape.org/, version: v3.520240101) were used to explore their biological significance and characterize diseases associated with AAbs, as performed previously (15, 16). We combined the increased autoantibodies from the three patients, according to the above description (*P* < 0.05 and fold change (FC) ≥ 2.0) in one list. Then, the list was mapped to the coding genes. Finally, the list of coding genes of the increased autoantibodies was performed GO enrichment analysis and KEGG pathway enrichment analysis via Metascape, respectively. GO or KEGG pathway terms with a *P*-value < 0.01, a minimum count of three, and an enrichment factor > 1.5 (the enrichment factor is the ratio between the observed counts and the counts expected by chance) were collected and grouped into clusters based on their membership similarities. Top 20 identified GO enrichment terms and KEGG pathway were presented.

## 3 Results

**Table 1** summarizes the clinical characteristics of the patients with ICI myocarditis included in this study. All patients received anti-programmed death-1 antibody monotherapy and developed myocarditis after 1-2 cycles of treatment. All patients were diagnosed with definite myocarditis based on definitions suggested by Bonaca et al. (17) and had tissue pathology suggestive of myocarditis on the endocardial biopsy. Two of the three patients had other concomitant myotoxicities (myositis with or without myasthenia gravis-like syndrome). All patients were treated with high-dose steroids, and one patient received additional intravenous immunoglobulin. Two patients were discharged alive, and one died of severe heart failure.

**Table 1:** Patient information.

Case	Age	Gender	Malignancy	Number of ICI cycles	Time to myocarditis onset from ICI start (days)	irAEs	Treatment	In-hospital outcome
1	73	M	RCC	2	29	Myocarditis, Myositis, MG-like syndrome	High-dose corticosteroids	Alive
2	57	F	NSCLC	1	24	Myocarditis	High-dose corticosteroids	Dead
3	80	M	Bladder cancer	1	29	Myocarditis, Myositis	High-dose corticosteroids, intravenous immunoglobulin	Alive

ICI, immune checkpoint inhibitor; irAE, immune-related adverse event; MG, myasthenia gravis; NSCLC, non-small-cell lung cancer; RCC, renal cell carcinoma.

**Figure 1:**
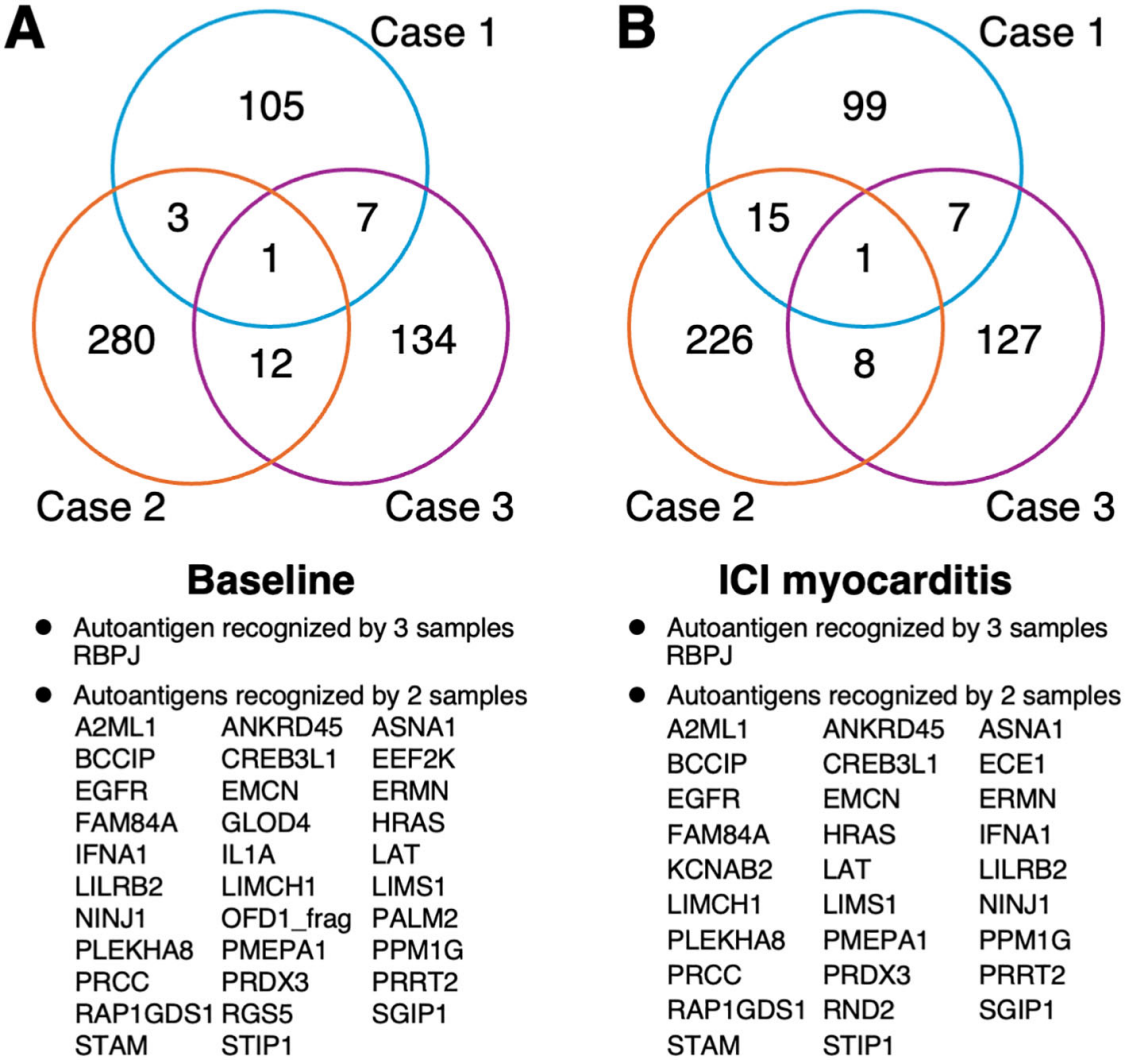
Venn diagram of the number of the immune-reactive autoantigens identified by human proteome microarray. **(A)** Before ICI treatment and **(B)** at the onset of ICI myocarditis **(B)**. ICI, immune checkpoint inhibitor.

To identify AAb signatures in ICI myocarditis, we performed AAb screening using a human proteome microarray containing approximately 17,000 unique full-length human proteins. We used sera collected before the initiation of ICI treatment (baseline) and immediately after the development of ICI myocarditis (before steroid treatment). At baseline, 116, 296, and 154 autoantigens reacted positively to IgG in the serum samples from Cases 1, 2, and 3, respectively (**Supplementary Table 1**). Among these proteins, one autoantigen (recombination signal binding protein for immunoglobulin kappa J region [RBPJ]) was recognized by all three samples, and 32 autoantigens were recognized by at least two of the three samples (**Figure 1A**). At the onset of ICI myocarditis, 122, 250, and 143 autoantigens reacted positively to IgG in the serum samples from Cases 1, 2, and 3, respectively (**Supplementary Table 1**). Among these proteins, RBPJ was recognized by all three samples. Additionally, 29 autoantigens were recognized by any two of the three samples (**Figure 1B**).

**Figure 2:**
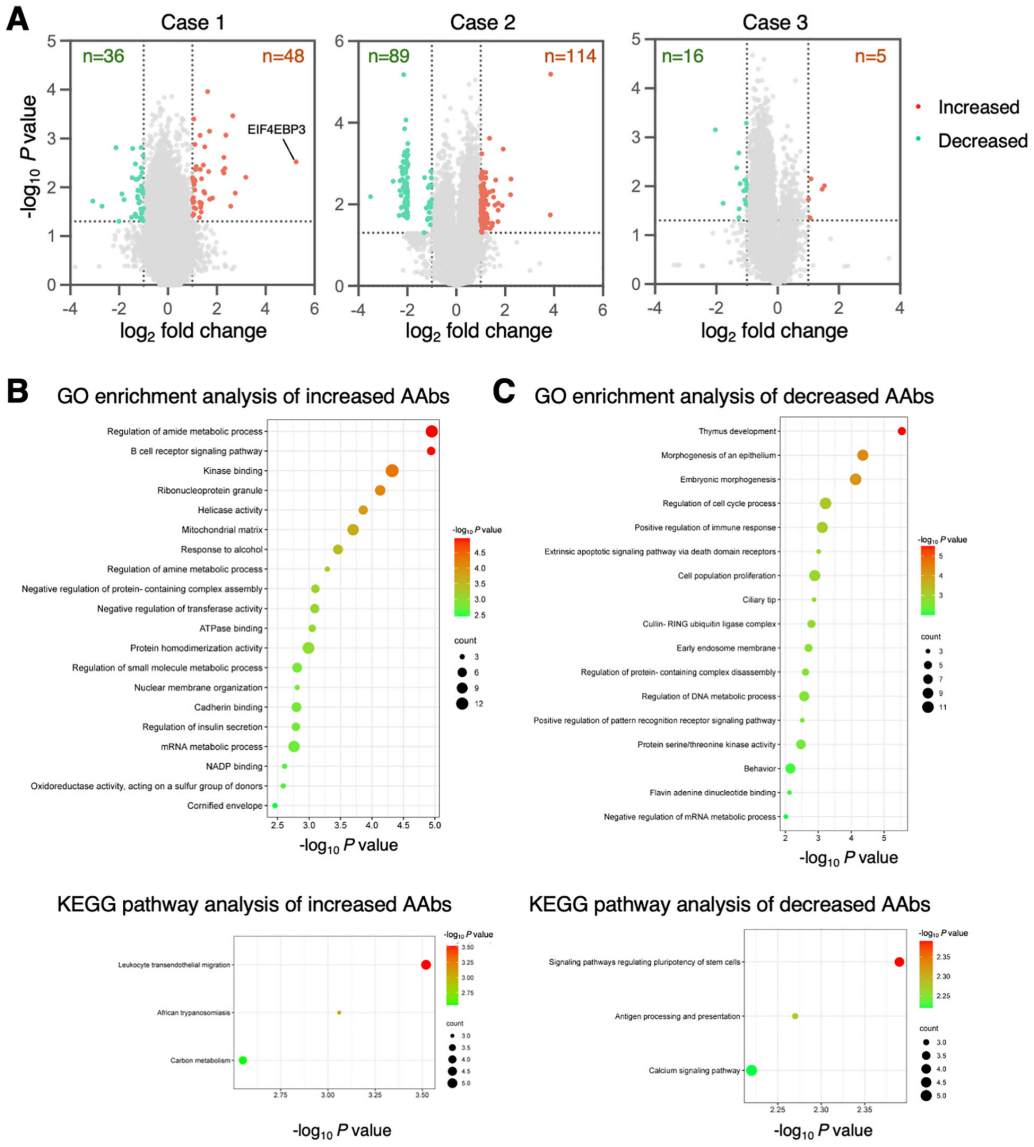
Differentially expressed autoantibodies in ICI myocarditis. **(A)** The volcano plots show the up- (red) or down-regulated (green) proteins at the onset of ICI myocarditis compared to baseline. **(B, C)** The top 20 GO enrichment terms and KEGG pathway categories involving upregulated **(B)** and downregulated IgG **(C)** antibodies. Negative log_10_-transformed *P*-value of significantly enriched GO terms and KEGG pathways (*P* < 0.01) are shown.

Next, we identified the specific and differentially expressed antibodies at the onset of ICI myocarditis. Antibodies with a change in antigen-antibody reactivity signal intensity of more than 2.0-fold or less than 0.5-fold (*P* < 0.05) between baseline and the onset of myocarditis were considered AAbs with different expression levels. The differentially recognized autoantigens are visualized in volcano plots (**Figure 2A**) and described in **Supplementary Table 2**. At the onset of ICI myocarditis, compared to baseline, 48, 114, and 5 autoantigens reacted more strongly with IgG in serum samples from Cases 1, 2, and 3, respectively, and 36, 89, and 16 autoantigens reacted less strongly, respectively. Among these, antibodies against EIF4EBP3 (eukaryotic translation initiation factor 4E [eIF4E] binding protein 3) were the most upregulated, showing a 38-fold increase (**Figure 2A**).

To better understand the biological relevance of the identified Abs, GO enrichment analysis was performed on their corresponding proteins. The results revealed that Abs increased during the development of ICI myocarditis and were profoundly involved in the regulation of amide metabolic processes and B-cell receptor signaling pathways (**Figure 2B**). In the KEGG pathway enrichment analysis, leukocyte transendothelial migration pathway was remarkably enriched (**Figure 2B**). In the AAbs that decreased during the development of ICI myocarditis, the most significantly enriched GO term was related to thymus development function (**Figure 2C**), and KEGG pathway enrichment analysis showed that the decreased AAbs were mainly involved in the signaling pathways regulating the pluripotency of stem cells and antigen processing and presentation (**Figure 2C**).

**Figure 3:**
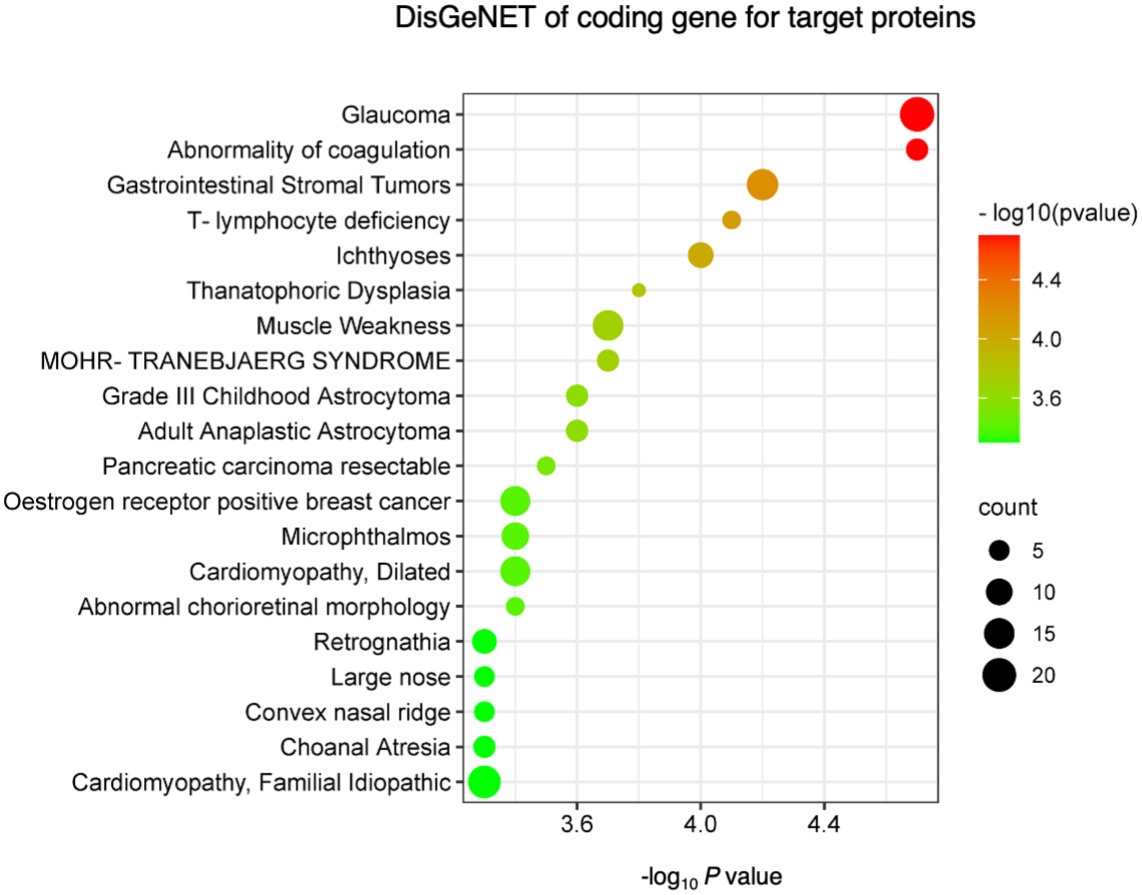
DisGeNET disease enrichment analysis. The top 20 diseases enriched in DisGeNETs were associated with upregulated and downregulated IgG antibodies. Negative log10-transformed P-value for significantly enriched diseases (P < 0.01) are shown.

Finally, we performed a DisGeNET disease enrichment analysis to explore the diseases in which these AAbs were involved (**Figure 3**). Enrichment diseases include T lymphocyte deficiency, muscle weakness, and dilated or familial idiopathic cardiomyopathy.

## 4 Discussion

This pilot study represents the first integrated analysis of serum AAb profiles in patients with ICI myocarditis, shedding light on potential novel candidate markers associated with the heightened risk of developing this and its pathogenesis.

Although RBPJ wasn’t involved in the pathways identified through GO or KEGG analysis, AAbs against RBPJ were detected in the sera of all patients with ICI myocarditis. RBPJ is a transcription factor that plays a crucial role in the Notch signaling pathway (18). The Notch signaling pathway mediated by RBPJ is involved in various cellular processes, including cell fate determination, differentiation, proliferation, and apoptosis, and plays critical roles in embryonic development, tissue homeostasis, and disease processes, such as cancer and heart diseases (19). There are few reports on AAbs targeting RBPJ, and their clinical significance remains unclear. Nickenig et al. reported an interesting finding that only 31% of healthy patients and 70.6% of patients with dilated cardiomyopathy (DCM) carry AAbs against RBPJ (20). They speculated that, because RBPJ is involved in cellular immortalization and exerts anti-apoptotic effects, increased anti-RBPJ AAbs may inhibit this growth-regulating feature of RBPJ in patients with DCM. Further research is needed to determine whether anti-RBPJ AAbs can be used as biomarkers for ICI myocarditis and whether they are involved in the pathogenesis of ICI myocarditis.

The AAbs that increased most remarkably during the development of ICI myocarditis were anti-EIF4EBP3 AAbs in Case 1. EIF4EBP3 is an eIF4E-binding protein that inhibits translation initiation by competing with eukaryotic translation initiation factor 4G for a common binding site on eIF4E (21–23). In our study, EIF4EBP3 was involved in the “regulation of amide metabolic process” term in the GO analysis results of coding genes for target proteins recognized by increased AAbs. Little is known about the role of EIF4EBP3 under normal and abnormal conditions, and its role of EIF4EBP3 in the heart is completely unknown.

Recent studies have shown that the presence of anti-AChR AAbs is more prevalent in patients with ICI myocarditis than in ICI-treated controls (11-36% vs 4%) (13, 24). Among the patients with ICI myocarditis, those with anti-AChR antibodies have a poorer prognosis than those without (13). However, we did not detect any anti-AChR antibodies in our study.

Enrichment analyses using GO and KEGG highlighted that B-cell receptor signaling, leukocyte transendothelial migration, and thymus development were among the most affected pathways. Enrichment analyses using DisGeNET revealed that proteins reacting with AAbs detected in patients with ICI myocarditis were associated with several diseases, including DCM and muscle weakness. This pilot study suggests that profiling serum AAbs could enhance our understanding of the pathogenesis of ICI myocarditis and facilitate the development of effective therapies, a more comprehensive search for AAbs in more cases is needed in the future.

Although our study offers new insights into the properties and changes in the AAb repertoire associated with ICI myocarditis, it has some limitations. The small sample size included in this study limits the generalizability of the present findings. In addition, we did not perform AAb profiling in ICI-treated controls (i.e., without myocarditis). Consequently, it is crucial to validate the identified Abs in larger, more diverse, and independent cohorts with appropriate controls to elucidate their roles in ICI myocarditis.

## 5 Conclusion

Human proteomic microarrays offer a powerful platform for the discovery of novel antibodies in patients with ICI myocarditis. This pilot study provides the first integrated analysis of serum AAb profiling in patients with ICI myocarditis and identifies novel candidate markers associated with an increased risk of developing ICI myocarditis and its pathogenesis. However, our results require further independent validation in clinical trials involving a larger number of patients. Further identification and characterization of AAbs are likely to hold significant implications for diagnostic and biomarker discovery, immune profiling, and the development of effective treatments.

## Data Availability

The raw data supporting the conclusions of this article will be made available by the authors, without undue reservation.
